# 

**Published:** 1857-03

**Authors:** 


### A WORD WITH OUR PATRONS.

We have now issued three numbers of the Dental Register, and we propose to give you some items of our brief experience. These issues have cost us a little more than five hundred dollars, while we have received on subscription but two hundred and eight, leaving a balance against us of more than three hundred dollars; and this, too, when we have the additional burden of paying the former proprietor for the Register, besides the expenses and loss of time incident to a change of location. This state of affairs does not arise from a limited list of subscribers, nor from any desire to read the Register without paying for it, but from the fact that each one feels that *three dollars* is a sum so insignificant that it matters but little when it is sent, and, as usual, a thing postponed is a thing neglected. There are not five subscribers on our list who would have thus delayed sending their dues had they supposed that, at the time we must pay for the issue of our third number, our receipts would barely pay for the first; but each one supposed that while he held back, others would remit.

We were quite surprised, on a careful review of Dr. Taylor's book, to find that his experience with the finances of the Register accords precisely with our own; but he was in a *full* tide of successful practice, in easy circumstances, good natured, and *very* patient, and, therefore, did not complain; but, as we profess to have but few of his gifts, and none of his graces, the like patience must not be expected of us.

In short, gentlemen, we again send bills to those who have not paid their subscription, and you will not consider us more selfish than our neighbors, when we state that we wish them to be paid. Do not wait for a convenient opportunity to send by private conveyance, but remit by mail at our risk.

To our friends who have paid their subscriptions we return our most cordial and hearty thanks, and especially to those who have aided in extending the circulation of the Register.

### NEW DENTAL COLLEGES.

Our readers will all be pleased to learn that the Dental Profession in Great Britain have, with great harmony, organized a Dental College. A

concise and satisfactory account of their proceedings may be seen in our miscellaneous department.

We also learn from the November number of the "Dental Recorder," that an attempt was being made to obtain a charter for a Dental College to be located in the city of New York, to be called "The New York College of Dental Surgery."

The editor of the Recorder expresses his views on the subject, at some length, and in the main, very much to the point. We are somewhat surprised, however, to hear of the gigantic proportions of the "Ohio Dental College Association," which he designates as the "largest Dental Society ever known." The child, when told that the coral islands are formed by living beings, no doubt thinks of *whales*, and on the same principle, works bearing witness, the editor, aware that the Association has purchased property and erected a beautiful and permanent College edifice, may conclude that it is at least "sizable."

The editor very correctly remarks that, "no one but a thoroughly educated and experienced Dentist can judge of the qualifications of a professional brother." If this position be correct, it follows that, to members of the profession alone should the selection of teachers of Dental Science be entrusted. We heartily coincide with the concluding remark of the editorial referred to: "We hope that our Legislature, should it see fit to charter a Dental College, will, with its usual good sense and practical judgment, place authority in the hands of those only who are competent to exercise it. Such parties are not to be found out of the Dental Profession."

The Ohio College of Dental Surgery is owned and controlled by an association, every member of which is a practical dentist, or is directly and *pecuniarily* interested in the prosperity of the Profession. This we regard as a pledge both of the integrity and the ultimate success of the Institution.

☞We have endeavored to be accurate in sending bills, but mistakes may have occurred. Should any one who has paid receive a bill, he will please notify us, and we will at once correct the mistake. If any one who has paid for the present volume is omitted in our published receipts, we make the same request of him.

☞ Just as our last form was going to press, we receivhd the proceedings of the "St. Louis Dental Society," we were, therefore, unable to place them under their appropriate head. This brief account of the society shows that our brethren in St. Louis, are not only alive, but wide awake. We have not yet received the *proceedings* of the "Cincinnati Dental Society." In fact, the society has not *proceeded.*

*Comparative Anatomy of the Teeth.*

### RECEIPTS FOR VOLUME X.

| | |
|---|---|
| Dr. A. Berry, | $3 00 |
| " H. H. Harvey, | 3 00 |
| " T. P. Abell, | 3 00 |
| " F. Searle, | 3 00 |
| " Sam'l Hapell, | 3 00 |
| " H. Garrett, | 3 00 |
| " St. T. Beale, | 3 00 |
| " W. W. Allport, | 3 00 |
| " A. L. Brown, | 3 00 |
| " P. A. Ferbrache, | 3 00 |
| " W. R. Webster, | 2 40 |
| " J. S. Knapp, | 3 00 |
| " L. M. Cochran, | 3 00 |
| " N. H. Drew, | 1 50 |
| " Sam'l Mallett, | 3 00 |
| " Chas. Bonsall, | 3 00 |
| " J. R. Walker, | 1 00 |
| " C. W. Spalding, | 3 00 |
| " E. J. Woodbury, | 3 00 |
| " M. H. Cooley, | 3 00 |
| " J. L. Springgate, | 3 00 |
| " H. A. Smith, | 3 00 |
| " J. B. Williams, | 3 00 |
| " H. Munro, | 3 00 |
| " D. L. Pratt, | 3 00 |
| " G. C. North, | 3 00 |
| " Geo. P. Newlin, | 3 00 |
| " A. G. Stipher, | 3 00 |
| " F. M. Dixon, | 3 00 |
| " J. Fleager, | 3 00 |
| " S. F. Compton, | 1 00 |
| " E. Hale, | 3 33 |
| " L. D. & J. S. Walter, | 3 00 |
| " John A. Doherty, | 3 00 |
| " John Hood, | 3 00 |
| " Dunham & Hale, | 3 00 |
| " J. R. James, | 3 00 |
| Dr. B. B. Alfred, | $3 00 |
| " Chs. H. Roberts, | 3 00 |
| " Overholser, | 3 00 |
| " A. S. Talbert, | 3 00 |
| " J. C. Whinery, | 3 00 |
| " J. H. Keith, | 2 00 |
| " J. A. Trousdale, | 3 00 |
| " E. C. Sloan, | 3 06 |
| " S. H. Harvey, | 3 00 |
| " D. Walters, | 3 00 |
| " J. O. Martin, | 3 00 |
| " Chas. E. Kells, | 3 00 |
| " E. G. Toof, | 3 00 |
| " J. H. Meredith, | 3 00 |
| " H. R. Hurd, | 3 00 |
| " Sam'l Rambo, | 3 00 |
| " Cowdery & Fogg, | 3 00 |
| " I. B. Branch, | 3 00 |
| " P. Knowlton, | 3 00 |
| " G. W. Keely, | 3 00 |
| " C. O. Cone, | 3 00 |
| " J. W. Fulton, | 3 00 |
| " H. N. Lewis, | 3 00 |
| " J. H. Hyde, | 3 00 |
| " W. L. Stovall, | 3 00 |
| " Elisha Townsend, | 3 00 |
| " Robt. Johnson, | 3 00 |
| " M. W. Wartman, | 3 00 |
| " Wm. C. Glines, | 3 00 |
| " Robt. Arthur, | 3 00 |
| " C. H. Quinlan, | 3 00 |
| " John W. Keely, | 3 00 |
| " J. W. Keyes, | 3 00 |
| " J. P. Ullery, | 3 00 |
| " F. A. Brewer, | 3 00 |
| " H. McCollum, | 3 00 |
| " W. B. Martin, | 3 00 |

### OHIO COLLEGE OF DENTAL SURGERY.

### Session of 1857-8.

---

The regular course of lectures in this institution, will commence on the first Monday of November, and close on the third Wednesday of February.

### FACULTY.

C. B. CHAPMAN, M. D.,
*Professor of Anatomy and Physiology.*

J. B. SMITH, M. D.,
*Professor of Pathology and Therapeutics.*

JAMES TAYLOR, D. D. S.,
*Professor of the Institutes of Dental Science.*

J. TAFT, D. D. S.,
*Professor of Operative Dentistry.*

JOSEPH RICHARDSON, D. D. S.,
*Professor of Mechanical Dentistry.*

GEORGE WATT, D. D. S.,
*Professor of Chemistry and Metallurgy.*

HENRY A. SMITH, D. D. S.,
*Demonstrator of Operative and Mechanical Dentistry.*

The Infirmary will be kept open during the summer and during the month of October special attention will be paid to operations before such of the class as can come in by that time. Those desiring *extra* opportunities for practice should avail themselves of this month's privileges.

To meet the wants of those engaged in practice, half course tickets are issued, thus enabling them to make out a full course of graduation without leaving their homes and practice for an entire session at one time.

Students should bring with them such instruments and text books as they may have on hand.

Candidates for graduation must have attended two full courses of Lectures, the last in this school, and must have enjoyed the advantages of a satisfactory Dental pupilage. Certificates of four years Dental Practice, or of one full course in any respectable Dental or regular Medical College, will be received as equivalent to the first course.

---

### TERMS OF ADMISSION.

The tickets are considered as cash, and when not paid for in advance, a note to the Dean of the Faculty, with approved security will be required.

| | |
|---|---|
| Tickets for the entire course, - - - - - - - | $100 00 |
| Matriculation Ticket, - - - - - - - | 5 00 |
| Diploma Fee, - - - - - - - | 25 00 |

Good and respectable board can be had from $2 50 to $4 per week.

GEORGE WATT, DEAN.

### BALTIMORE COLLEGE OF DENTAL SURGERY.

**SIXTEENTH ANNUAL SESSION, 1856-57.**

**FACULTY.**

CHAPIN A. HARRIS, D. D. S., M. D.
*Principles of Dental Surgery.*
THOMAS E. BOND, M. D.
*Therapeutics and Materia Medica.*
WASHINGTON R. HANDY, M. D.
*Anatomy and Physiology.*
PHILIP H. AUSTEN, D. D. S., M. D.
*Mechanical Dentistry.*
EDWARD MAYNARD, D. D. S., M. D.
*Theory and Practice of Dental Surgery.*
REGINALD N. WRIGHT, M. D.
*Chemistry and Metallurgy.*
CHRISTOPHER JOHNSTON, M. D.
*Microscopical and Comparative Anatomy of the teeth.*
GEORGE W. NEIDICH D. D. S.
*Demonstrator of Dental Surgery.*

The Seventeenth Annual Session of the Baltimore College of Dental Surgery, will open on the first of October, and close on the third of March. The Regular Course of Lectures will commence on the third of November.

The month of October will be devoted to Practical Dentistry and Anatomical Dissections.

The large practice of the Infirmary offers to the Student supeirior facilities for the acquirement of a *practical* knowledge of Medical, Surgcal, and Mechanical Dentistry.

Professors' Tickets, $110; Infirmary Ticket, $10; Dissection Ticket. (optional) $10; Matriculation Fee, $5; Diploma Fee, $30.

Good Board may be procured at from $3 50 to $4 00 per week. For further nformation address.

P. H. AUSTEN, *Dean of the Faculty.*

*No.* 76 *Sharp Street.*

---

### DENTAL DEPOT.

### JOHN KLEIN,

MANUFACTURER OF

### PORCELAIN TEETH,

### Gold & Tin Foil, Gold & Silver Plate, Wire, Spiral Springs

INSTRUMENTS, &c., &c.

*No.* 16½ *North Eighth street, Philadelphia, Pa.*

Constantly on hand a general assortment of Improved Plate, Pivot, Molar, Bicuspids, and Gum Teeth, at reduced prices; together with an assortment of Dental Instruments, Materials, Tools, Lathes, Wheels, Molding Flasks, Gold and Tin Foils, Files, Blow Pipes, Lamps, Head Rests, &c., &c. All Orders promptly filled.

TENTH VOLUME

OF THE

### DENTAL REGISTER OF THE WEST.

EDITED BY

**J. TAFT and G. WATT.**

TERMS: - - - - - - - - $3 PER ANNUM, IN ADVANCE.

**Advertisements Inserted as Follows:**

One page, per annum....................$25 00
One-half page, per annum.................... 15 00
Quarter page, per annum.................... 10 00
Card (five lines or less),.................... 5 00,

Shorter periods than one year, by special arrangement.

☞ Contributions to the pages of the *Register* respectfully solicited.

Exchanges and correspondents will please address, "Dental Register," or "Taft & Watt, Cincinnati, Ohio."

